# Preferences for Delivering Brief Alcohol Intervention to Risky Drinking Parents in Children’s Social Care: A Discrete Choice Experiment

**DOI:** 10.1093/alcalc/agac018

**Published:** 2022-04-21

**Authors:** R McGovern, T Homer, E Kaner, D Smart, L Ternent

**Affiliations:** Population Health Sciences Institute, Newcastle University, Newcastle upon Tyne, NE2 4AX, UK; Population Health Sciences Institute, Newcastle University, Newcastle upon Tyne, NE2 4AX, UK; Population Health Sciences Institute, Newcastle University, Newcastle upon Tyne, NE2 4AX, UK; Population Health Sciences Institute, Newcastle University, Newcastle upon Tyne, NE2 4AX, UK; Population Health Sciences Institute, Newcastle University, Newcastle upon Tyne, NE2 4AX, UK

## Abstract

**Aims:**

Many parents in contact with children’s social care services misuse alcohol however do not meet the threshold for specialist alcohol treatment, and typically do not receive appropriate support for their needs. Brief alcohol interventions have been found to be effective in healthcare settings, however, it is unknown whether the brief intervention structure delivered within health settings would transfer well into children’s social care. This paper aims to examine the characteristics of brief intervention for alcohol misusing parents which social care practitioners consider to be important and acceptable to implement in this sector.

**Methods:**

We assessed preferences for, and acceptability of, brief alcohol intervention with parents in contact with children’s social care using a discrete choice experiment. We recruited 205 children’s social care practitioners from London and the North East of England. Data were analysed using mixed logit which accounted for repeated responses.

**Findings:**

Six attributes showed statistically significant coefficients, suggesting that a brief intervention with these attributes would encourage implementation. These were: level of alcohol-related risk targeted; intervention recipient; timing of intervention; duration of sessions; number of sessions and intervention structure. The attribute of most importance identified based on the attribute with the largest coefficient in the conditional logit model was risk level.

**Conclusions:**

Brief alcohol interventions delivered to parents in social care should focus on the impact upon children and the wider family, they should be a flexible part of on-going casework and should be more intensive and less structured.

## Teaser

**Short Summary:** BAIs in health settings typically following universal screening, are delivered in a single session and provide simple structured advice. In children’s social care BAIs need to be targeted at parents whose alcohol use is causing observable problems within the family and should be more intensive and less structured.

## INTRODUCTION

In the UK, it is estimated that 478,000 children lived with a parent who misuses alcohol or drugs in 2019–2020; a rate of 40 per 1000 children (Children's Commissioner, 2020) and 10.5% of children aged under 17 years in USA (7.5 million) live with at least one alcohol misusing parent (Lipari and Van Horn, 2017), with substantial risk for both the parent (Lim et al., 2012) and the child (Canfield et al., 2017). Due to the potentially negative impact on the child, parental alcohol misuse is often identified as a risk factor in child welfare and child protection assessments (Hafekost et al., 2017) and care proceedings (Raitasalo et al., 2015) undertaken by child social care services. However many parents who misuse alcohol do not meet the threshold for specialist alcohol treatment (Forrester and Harwin, 2006). A major review of UK child protection services highlighted the importance of preventive rather than reactive services as being more effective in improving child welfare (Munro, 2011). Despite this, there is no established preventative intervention for parental alcohol misuse (McGovern et al., 2021). Further, there remains uncertainty about who is best placed to respond to these lower level alcohol needs and what severity and nature of alcohol-related concern merited intervention from social care (Hafford-Letchfield et al., 2017). Typically, this results in many alcohol misusing parents receiving no intervention to reduce alcohol-related risk or in their referral into specialist treatment, despite both parents and treatment providers considering this to be inappropriate to their needs (Forrester and Harwin, 2006). Given the close proximity of social care practitioners to alcohol misusing parents within their routine practice, it has been suggested that the delivery of brief alcohol interventions within social care maybe a useful approach to responding to the alcohol-related needs of this population (Schmidt et al., 2014).

There is a large amount of high-quality evidence which has accumulated to support of brief alcohol interventions delivered within a healthcare setting with alcohol misusing adult patients (Kaner et al., 2016). Brief alcohol interventions vary from simple structured advice to unstructured counselling (National Institute for Health and Clinical Excellence, 2010), ranging from 5 to 60 min in a single session or a series of related sessions (not exceeding five sessions) (Kaner et al., 2016). Patients receiving brief alcohol intervention report drinking less alcohol at 12 months follow-up when compared to minimal or no intervention (Kaner et al., 2016), with no-significant additional effect from extended interventions (comprising of either more than five sessions or >60 min in total) (Kaner et al., 2016). Most of this evidence has been in primary care, however there is some evidence of effect in other settings including in emergency departments (McQueen et al., 2011) and with other populations such as pregnant women (Marais et al., 2011). This evidence-base informed National Institute for Health and Care Excellence recommendations that brief alcohol interventions should be implemented in a range of settings in the UK including social care (National Institute for Health and Clinical Excellence, 2010). However, there is a paucity of studies examining the effectiveness of alcohol screening and brief interventions within social care settings or specifically its use with parents referred to children’s social care to support and safeguard the child (McGovern et al., 2018). Furthermore, little is understood about the acceptability of brief interventions to the social care workforce or the optimum implementation approach. In particular, it is unknown whether the brief intervention structure delivered within health settings would transfer well into children’s social care (Hafford-Letchfield et al., 2017), wherein there are different working practices and priorities.

Brief alcohol interventions may promote the parent’s ability to link their drinking with adverse experiences and the risk of negative outcomes for their child, as well as to themselves, and therefore have the potential to replicate the ‘teachable moment’ found to be conducive of behaviour change following the delivering of brief alcohol interventions within other settings (Babor and Grant, 1992). However, children and families in contact with children’s social care often experience a range of complex problems. Children’s social care has responsibilities to protect or remove children from situations of significant harm, and therefore interactions with social care practitioners can be experienced as threatening by parents, resulting in their resistance to related intervention (Forrester et al., 2012). Practitioners report that raising the issue of alcohol may heighten this threat and jeopardize any already difficult relationship (Galvani et al., 2013; Hafford-Letchfield et al., 2017). It has also been noted that the limited guidance for social care practitioners in how to raise and respond to the alcohol needs of social care users is a barrier to intervening, particular in safeguarding contexts (Hafford-Letchfield et al., 2017). The aim of the current study is to examine the characteristics of brief intervention for alcohol misusing parents which children social care practitioners consider to be important and acceptable within this setting.

## METHODS

We assessed preferences for, and acceptability of, brief alcohol intervention with parents in contact with children’s social care using a discrete choice experiment (DCE). DCEs requires respondents to make choices between hypothetical alternatives. Each alternative has a common set of characteristics (attributes) however, the value (level) of the attributes vary. The results of the DCE can therefore provide information on the relative importance of the different attributes and their associated levels. The data can be used to consider potential trade-offs and acceptability of different intervention configurations. This information could then be used by decision-makers within children’s social care to inform the most acceptable configuration of brief alcohol intervention to be delivered to parents by social care practitioners. DCEs are widely used in health care (Soekhai et al., 2019) and have specifically been used to look at preferences and acceptability of alcohol interventions (Pechey et al., 2014). They are based upon random utility theory, which assumes economic rationality and utility maximization (Ryan, 2004). The assumption being that when presented with a number of options (choice sets) respondents will choose the alternative that maximizes their utility (i.e. satisfaction). Best practice guidelines in the design and conduct of DCE were followed throughout (Ryan et al., 2008). Ethical approval for the study was obtained from the Health Research Authority, Social Care Research Ethic Committee (ref 16/IEC08/0037).

### Attributes and levels

We used an established staged process recommended by Helter and Boehler (2016) to develop the attributes for our DCE (Helter and Boehler, 2016). Within stage one we reviewed the findings of two systematic literature reviews examining barriers and facilitators to implementing brief alcohol interventions in health and community settings (Johnson et al., 2011; Derges et al., 2017). The systematic literature reviews reported that a simple means of identifying alcohol-related need, training and institutional support to be important to the implementation of brief interventions. Additionally, it was reported that practitioners often found it difficult to find time to implement brief alcohol interventions due to workload pressure and competing priorities. These factors were further examined in three focus groups with social care practitioners (*n* = 21 participants) and one focus group with drug and alcohol practitioners (*n* = 7 participants). The focus groups were conducted by the lead author (RM) and examined practitioner’s views on providing an intervention to parents who misuse alcohol. The focus groups highlighted variation in social care practitioners’ sense of legitimacy in intervening around alcohol misuse, the sensitivity of discussing alcohol use within a context of child welfare; the importance of building trusting relationships; practice emphasis upon involving the child and the need for often intensive and prolonged intervention to meet the complex needs of vulnerable children and families who are in contact with social care. The findings of the systematic literature reviews and the focus groups were combined to produce a longlist of 19 potential attributes.

Within stage two we coded the focus group data according to the 19 attributes. Further analysis demonstrated that some of the attributes within the systematic literature reviews and those identified within the focus group transcripts reported on aspects of brief alcohol intervention implementation which were related and could be grouped resulting in a reduced (refocused) list of 15 attributes. Three potential attributes of building a trusting relationship, alcohol as a sensitive topic and the safeguarding context were subsequently combined to form an attribute which examined the timing of the intervention.

Within a further stage, seven attributes were rejected from the design by the research team due to a lack of importance being identified within the focus groups or because they were deemed to be inappropriate attributes on the basis that they could not be influenced by intervention design (for example practitioner attitude). The remaining eight key attributes were included within our design; all with either two or four levels are presented in Table 1.

**Table 1:** Attributes and levels

Attribute	Levels
The nature of alcohol-related risk	All parents regardless of risk^*^
	Any risky drinking parent
	Alcohol risk that impacts upon the child and family
	Alcohol is main safeguarding concern
Intervention recipient	Parent only^*^
	Parent and child
Timing of intervention	Assessment phase^*^
	During on-going casework
Session duration	10 min^*^
	20 min
	40 min
	60 min
Number of sessions	Single session^*^
	Two sessions
	Three sessions
	Six sessions
Structure	Information leaflet^*^
	Structured advice
	Semi-structured advice
	Counselling
Organization support	Supervision^*^
	Supervision plus organization performance monitoring
Training	Half-day^*^
	Full-day

^*^Reference level.

The attributes and associated levels provide 4096 possible combinations therefore, a full factorial design was not feasible. To reduce this whilst maintaining our ability to estimate all main effects we used a D-efficient design. The final design chosen consisted of 80 possible scenarios using the Ngene design software (ChoiceMetrics, 2017). The design chosen was the most efficient design that minimized the standard errors.

### Questionnaire design

**Figure 1:**
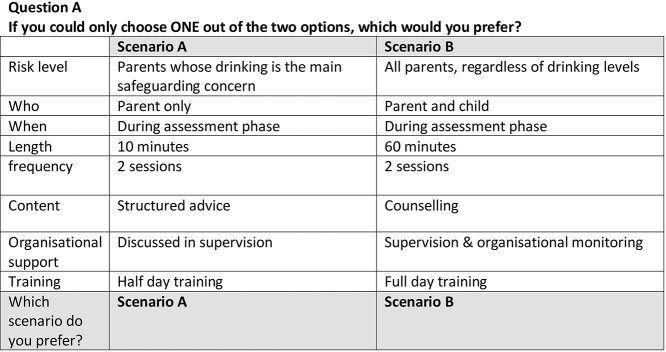
Choice set example.

To minimize burden, the 80 possible scenarios were randomly ‘blocked’ into four groups. Participants were provided with one of four blocked paper questionnaires to complete, each containing 20 scenarios with two alternative choice sets (Fig. 1). Participants were asked to select their preferred scenario between the two possible combinations. Each participant was also asked to provide sociodemographic and other relevant details about their practice role as well as Likert scale questions relating to the acceptability and feasibility of delivering brief alcohol interventions within their routine practice.

### Participants

Researchers attended a conference event aimed at the children’s social care workforce on the topic of parental alcohol misuse and gave a presentation on brief alcohol interventions before inviting practitioners to complete the DCE whilst in attendance at the event. Completed questionnaires were collected at the end of the event. Additionally, the researchers attended team meetings organized within children’s social care services. This approach allowed researchers to monitor the distribution of completed questionnaires across the four blocks, and rebalance variation. Researchers gave a general introduction to brief alcohol interventions, explained the attributes and levels in order to promote participant understanding (Coast et al., 2012) and provided guidance on how to complete the questionnaire.

### Analysis

Data were analysed in STATA (StataCorp, 2017). Sociodemographic data were analysed using descriptive statistics. Conditional regression logistic models were used to analyse respondents’ preferences for each of the scenarios chosen and to quantify the relative importance of the attributes and levels (McFadden, 1973). A positive coefficient represented a preference or utility associated with an attribute level in comparison to the reference level. A negative coefficient represented a disutility associated with an attribute level in comparison to the reference level. If the *P*-value for the coefficient was ≤0.05 the preference for the attribute level was statistically significantly different from zero. A reference level was chosen for each attribute and all attribute levels are compared to the reference level (see Table 1). It was anticipated that preferences for the attributes and levels would differ based on participants’ years’ experience in health and social care practice, as has been shown in research examining alcohol and drug interventions delivered by non-specialists in novel settings (Rouhani et al., 2019). Consequently, we analysed data comparing highly experienced and less experienced practitioners. In a sensitivity analysis a mixed logit model was undertaken to identify any potential heterogeneity within the data. Finally, the relative attribute importance (RAI) scores were estimated to facilitate comparison between the attributes. The RAI scores estimate the relative importance of each attribute in the intervention design in relation to the most important attribute.

## FINDINGS

In total, 205 practitioners responded to the DCE. The mean age of participants was 42.95 (SD 10.67) years. Most were female (88%) and had a mean of 12.63 (SD 9.23) years of experience in health and social care practice.

The results of the conditional logit are presented in Table 2, Six attributes showed statistically significant coefficients, suggesting that a brief intervention with these components would encourage implementation. These were: level of alcohol-related risk targeted; intervention recipient; timing of intervention; duration of sessions; number of sessions and intervention structure. Practitioners preferred to deliver brief interventions to parents where there was a clearly known or indicated risk rather than universal intervention to all parents. The strongest preference was for intervening with parents where their alcohol use was identified as impacting upon the child and/or the family, followed by ‘any risk’ then ‘alcohol as the primary safeguarding concern’. Participations stated preference in involving the child in the delivery of the intervention with parent rather than to the parent only. There was a preference for the intervention to be delivered within on-going casework within an established relationship with the family rather than during the in assessment stage, wherein the practitioner was developing an understanding of risk. More intensive interventions were preferred over very brief input, with the strongest preference being for six sessions (each lasting 40 min), with no preference for two or three sessions over a single session of brief intervention. Participants preferred all interactional approaches such as semi-structure counselling to delivering brief alcohol interventions over the provision of an information leaflet, however the strongest preference was for interventions with a semi-structured design. The level of organizational support and training was found to have little influence on practitioners’ implementing brief alcohol interventions. Table 3 summaries the optimal brief alcohol intervention based on practitioners’ preferences.

**Table 2:** DCE results

Attribute level	Co-eff (SE)	*P*-value	Interpretation
Risk level—base = all parents regardless of drinking levels			
Any risky drinking parent	0.428 (0.10)	0.000	Participants prefer to deliver the intervention for all of these risk levels and have the strongest preference to intervene when parents drinking impacts the child/family.
Parents whose drinking impacts upon child/family	0.712 (0.09)	0.000	
Parents whose drinking is the main safeguarding concern	0.317 (0.05)	0.000	
**Who—base = parent only**			
Parent and child	0.155 (0.04)	0.000	Participants prefer to deliver the intervention to the parents & child.
When—base = during assessment phase			
During on-going casework	0.129 (0.04)	0.001	Participants prefer to deliver the intervention as part of on-going casework.
**Length—base = 10 min**			
20 min	0.291 (0.08)	0.001	Participants preferred delivering the intervention for >10 min. With the strongest preference for 40 min.
40 min	0.311 (0.07)	0.000	
60 min	0.287 (0.05)	0.000	
**Frequency—base = 1 session**			
2 sessions	0.0879 (0.08)	0.266	Participants did not have a preference for 2 or 3 sessions compared to 1 session. However participants preferred 6 sessions to 1 session.
3 sessions	0.144 (0.08)	0.072	
6 sessions	0.202 (0.05)	0.000	
**Content—base = leaflet**			
Structured advice	0.268 (0.10)	0.005	Participants preferred all 3 ways to deliver the content compared to a leaflet and they had the strongest preference for semi-structured discussions.
Semi-structured discussion	0.292 (0.09)	0.001	
Counselling	0.237 (0.05)	0.000	
**Organizational support—base = discussed in supervision**			
Supervision and organizational monitoring	−0.00829 (0.04)	0.819	Participants had no preference for how organizational support was provided.
**Training—base = half-day**			
Full-day	−0.0517 (0.03)	0.138	Participants had no preference for the length of training provided.
Alternative A	−0.0472 (0.03)	0.150	Participants had no preference for choosing on alternative over another, i.e. no alternative bias.

**Table 3:** Optimal intervention

• Intervene when parents’ drinking is impacting the child and/or family.
• Intervention should be delivered to both the parents and the child.
• The intervention should be delivered as part of on-going casework.
• The length of the intervention should be 40 min.
• The intervention should be delivered for 6 sessions.
• The intervention should be delivered as a semi-structured discussion.

The sample was split into two groups and the conditional logit was run on a sample of highly experienced practitioners (10 years and more in practice) and on a sample of those who have less experience (<10 years in practice). These regression results are included as supplementary material.

This analysis showed that experienced practitioners preferred to deliver the intervention with both the parent and child and to opt for shorter duration sessions and interventions providing structured intervention. Practitioners with <10 years of experience preferred a more intensive intervention consisting of six session of 60 min each. Additionally, less experienced workers stated a preference for semi- or unstructured interventions. The sensitivity analysis estimating participants’ preferences using a mixed logit model produced similar results to the conditional logit (see Supplementary Material Table 1). However, we were able to identify heterogeneity within the sample.

**Figure 2:**
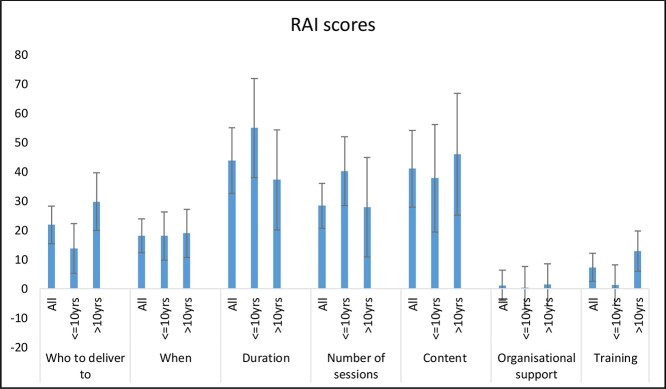
RAI scores.

The attribute of most importance identified based on the attribute with the largest coefficient in the conditional logit model was risk level. Figure 2 illustrates the relative attribute importance based on the conditional logit models discussed above, using a normalization based on the risk attribute. In the primary analysis, with the whole sample, duration was the next important attribute and was 44% as important as risk level to participants. This was consistent for those with <10 years of experience. However, the content of the intervention was more important to those more experienced as it was 46% as important as risk level.

## DISCUSSION

Our findings suggest that brief alcohol interventions in children’s social care must be adapted to the central function of supporting children and families. Specifically, this requires a move away from screening and identification based primarily on health-related risk (Saunders et al., 1993) to one that seeks to identify and prevent family-related risk. However, there are currently no screening tools which have been developed for use with parents or that are focused upon the risk parental alcohol misuse presents to their child(ren). Our own on-going pilot feasibility trial examining brief alcohol interventions delivered to parents in contact with social care (McGovern et al., 2018) utilized AUDIT-C. This tool, which asks only about consumption and not impact, was used to overcome the perceived sensitivity of asking parents about alcohol within a context of safeguarding children. Our theory of change highlighted an assumption that parents may be less inclined to disclose alcohol use if they felt their ability to parent is under question. Although screening based only on consumption may encourage the disclosure by parents, the findings of our DCE suggests that such an approach may not encourage social care practitioner’s to administer the tool as it does not seek to identify those parents whose drinking impacts upon the child. A move away from universal screening approaches recommended within health settings (Coulton et al., 2017) towards targeted screening based upon observable problems within the family linked to alcohol may be necessary within a social care setting.

The findings of this DCE suggest that practitioners considered it preferable to intervene with parents around alcohol as part of on-going casework. This is most likely due to the sensitivity of the topic and the sense that practitioners first need to build a trusting relationship. A more flexible approach regarding when to introduce the topic of alcohol would have the additional benefit of affording the practitioner the opportunity to identify alcohol as an issue for the family, and therefore connect alcohol use with child welfare priorities and reduce practitioner resistance to address the topic. In such a situation, screening is less about identifying risk and more about giving practitioners a structure by which to open a conversation with a parent about their alcohol use, as previous research has shown that social care practitioner often find it difficult to address alcohol use with parents (Galvani et al., 2013). Further, asking the parent questions about their alcohol in a situation wherein alcohol has already been identified as an area of concern may result in an approach which is more focused on the parent beginning to consider their alcohol misuse. Indeed research has found that being asked and answering questions about one’s own alcohol use can produce ‘screening reactivity’ and result in a reduction in alcohol consumption (Walters et al., 2009).

There was a clear preference for intervention that involves the child, particularly in more experienced practitioners. Children have been found to be a major motivator for parents to change their substance using behaviour (Frazer et al., 2019). This is in line with social care practice which emphasizes the importance of the voice of the child (O'Reilly and Dolan, 2016) and has the potential to enhance the parents’ understanding of the impact upon the child and therefore may motivate behaviour change. However, children may not be fully aware of their parents’ drinking levels, and well-intended efforts to include the child may have adverse effects, including making the child feel shame or embarrassment about their parent’s alcohol consumption (O'Shay-Wallace, 2020). The appropriateness of involving the child in the intervention may be affected by the level of exposure the child has to the parent’s alcohol misuse, the risks associated with their pattern of alcohol use and the age of the child. Further, a recent meta-analysis found that parents did not reduce the frequency of their alcohol use when children were involved in psychosocial intervention sessions and it was suggested that the presence of the child may affect the parent’s engagement with the intervention (McGovern et al., 2021).

Evidence from other settings (predominately primary health care) suggests that there is no additional benefit of extended intervention for people who misuse alcohol, with single session interventions, or multiple sessions of no >60 min in total (Kaner et al., 2016). However, our findings suggest that social care practitioners may prefer a more intensive and frequent intervention. Here practitioners’ reported the strongest preference for six sessions of brief alcohol intervention each lasting 40 min; an accumulative duration four times longer than that recommended in health. A number of factors could explain this disparity. Most brief alcohol interventions are delivered by generalist practitioners in primary health care where practitioner–patient consultations are very brief; typically 9.2 min in the UK (Royal College of General Practitioners, 2019). These appointments are often one-off appointments in response to a specific health concern. In contrast, social care–parent interactions tend to be longer appointments and often occur over extended periods of time. Additionally, families often present to social care services with complex needs and multiple vulnerabilities. Practitioners may perceive these needs as requiring more than single-session brief interventions, when the problems they seek to address are long-standing and within disadvantaged populations. Further, the extended brief intervention may support the on-going development of a trusting relationship wherein the parent may increasingly feel able to discuss their alcohol use in an open manner. One of the challenges with more intensive approaches to brief alcohol interventions however is whether the parent themselves would consider the intervention to be excessive to their needs. Those parents who are drinking above the recommended low-risk levels but are not currently experiencing harm related to their drinking may not require or recognize the need for more intensive brief intervention. This may further point to the need to target alcohol brief interventions in children’s social care with parents where harm is known. A further challenge maybe overall practitioner acceptance. When exploring heterogeneity our analyses showed that more experienced practitioner’s preferred shorter duration. It is possible that this could be explained by higher workload and also related fatigue which is likely to present a challenge to implementing an extended brief intervention within a social care setting.

This study is the first to examine preferred design of an alcohol brief intervention in social care, and therefore makes an important contribution to the field. Our ability to examine interactions between attributes was limited by our sample size. Optimal sample size requirements for the limited dependent variable models of the nature estimated in DCEs depend on knowledge of the true choice probabilities, which are not known prior to undertaking this research. However previous DCE studies have shown that robust choice models can be estimated from sample sizes between 50 and 100 respondents (Adams et al., 2015), with most DCEs falling between 100 and 300 participants (Marshall et al., 2010). Although our findings provide important insights into the preferences of social care practitioners in the delivery of brief alcohol interventions, further research is needed to examine the preferences of risky drinking parents who may receive brief alcohol interventions. This will enable the development of an intervention which is most likely to be implemented by practitioners and acceptable to parents. Additionally research examining the effectiveness of this intervention and its applicability within the real-world setting would advance knowledge in the field and provide the evidence necessary to inform practice.

## CONCLUSION

The findings of our DCE suggest that the brief intervention structure delivered within health settings may not simply transfer into social care. Our work indicates that brief alcohol interventions delivered in social care with parents should target parents where there is known alcohol risk to the child and/or family, they should be a flexible part of on-going casework and should be more intensive and less structured.

## Supplementary Material

Supplementary_material_agac018
