# Supplementary material for: Preferences for Delivering Brief Alcohol Intervention to Risky Drinking Parents in Children’s Social Care: A Discrete Choice Experiment
Source: Alcohol Alcohol. 2022 Apr 21;57(5):615–21. doi: 10.1093/alcalc/agac018 (PMC9465525; doi:10.1093/alcalc/agac018)
Supplement: Supplementary_material_agac018 [file supplementary_material_agac018.docx]

Supplementary Material

Table 1 Mixed Logit results

|  | (1) | (2) | (3) | (4) | (5) | (6) | (7) | (8) |
| --- | --- | --- | --- | --- | --- | --- | --- | --- |
| VARIABLES | mixogit | mixogit | mixogit | mixogit | mixogit | mixogit | mixogit | mixogit |
|  |  |  |  |  |  |  |  |  |
| altwhowhere_riskyparent_d | 0.612*** | 0.217 | 0.612*** | 0.217 | 0.612*** | 0.217 | 0.612*** | 0.217 |
|  | (0.125) | (0.190) | (0.125) | (0.190) | (0.125) | (0.190) | (0.125) | (0.190) |
| altwhowhere_impactchild_d | 1.046*** | 0.615*** | 1.046*** | 0.615*** | 1.046*** | 0.615*** | 1.046*** | 0.615*** |
|  | (0.130) | (0.132) | (0.130) | (0.132) | (0.130) | (0.132) | (0.130) | (0.132) |
| altwhowhere_safeguarding_d | 0.382*** | 0.828*** | 0.382*** | 0.828*** | 0.382*** | 0.828*** | 0.382*** | 0.828*** |
|  | (0.0867) | (0.0959) | (0.0867) | (0.0959) | (0.0867) | (0.0959) | (0.0867) | (0.0959) |
| altwhich_parentandchild_d | 0.0929 | 1.076*** | 0.0929 | 1.076*** | 0.0929 | 1.076*** | 0.0929 | 1.076*** |
|  | (0.0854) | (0.0829) | (0.0854) | (0.0829) | (0.0854) | (0.0829) | (0.0854) | (0.0829) |
| altwhen_ongoing_d | 0.174*** | -0.244*** | 0.174*** | -0.244*** | 0.174*** | -0.244*** | 0.174*** | -0.244*** |
|  | (0.0491) | (0.0779) | (0.0491) | (0.0779) | (0.0491) | (0.0779) | (0.0491) | (0.0779) |
| altduration_20mins_d | 0.427*** | -0.108 | 0.427*** | -0.108 | 0.427*** | -0.108 | 0.427*** | -0.108 |
|  | (0.105) | (0.125) | (0.105) | (0.125) | (0.105) | (0.125) | (0.105) | (0.125) |
| altduration_40mins_d | 0.433*** | -0.217* | 0.433*** | -0.217* | 0.433*** | -0.217* | 0.433*** | -0.217* |
|  | (0.0962) | (0.121) | (0.0962) | (0.121) | (0.0962) | (0.121) | (0.0962) | (0.121) |
| altduration_60mins_d | 0.402*** | 0.641*** | 0.402*** | 0.641*** | 0.402*** | 0.641*** | 0.402*** | 0.641*** |
|  | (0.0792) | (0.103) | (0.0792) | (0.103) | (0.0792) | (0.103) | (0.0792) | (0.103) |
| altsessions_2_d | 0.0840 | 0.149 | 0.0840 | 0.149 | 0.0840 | 0.149 | 0.0840 | 0.149 |
|  | (0.109) | (0.139) | (0.109) | (0.139) | (0.109) | (0.139) | (0.109) | (0.139) |
| altsessions_3_d | 0.124 | -0.166 | 0.124 | -0.166 | 0.124 | -0.166 | 0.124 | -0.166 |
|  | (0.106) | (0.106) | (0.106) | (0.106) | (0.106) | (0.106) | (0.106) | (0.106) |
| altsessions_6_d | 0.256*** | 0.791*** | 0.256*** | 0.791*** | 0.256*** | 0.791*** | 0.256*** | 0.791*** |
|  | (0.0885) | (0.114) | (0.0885) | (0.114) | (0.0885) | (0.114) | (0.0885) | (0.114) |
| altcontent_structured_d | 0.310*** | 0.0654 | 0.310*** | 0.0654 | 0.310*** | 0.0654 | 0.310*** | 0.0654 |
|  | (0.119) | (0.140) | (0.119) | (0.140) | (0.119) | (0.140) | (0.119) | (0.140) |
| altcontent_semistructured_d | 0.354*** | 0.234* | 0.354*** | 0.234* | 0.354*** | 0.234* | 0.354*** | 0.234* |
|  | (0.113) | (0.134) | (0.113) | (0.134) | (0.113) | (0.134) | (0.113) | (0.134) |
| altcontent_counselling_d | 0.273*** | 0.638*** | 0.273*** | 0.638*** | 0.273*** | 0.638*** | 0.273*** | 0.638*** |
|  | (0.0764) | (0.119) | (0.0764) | (0.119) | (0.0764) | (0.119) | (0.0764) | (0.119) |
| altorgsupport_casework_monitor_d | -0.00218 | 0.0977 | -0.00218 | 0.0977 | -0.00218 | 0.0977 | -0.00218 | 0.0977 |
|  | (0.0458) | (0.0765) | (0.0458) | (0.0765) | (0.0458) | (0.0765) | (0.0458) | (0.0765) |
| alttraining_fullday_d | -0.0546 | 0.0210 | -0.0546 | 0.0210 | -0.0546 | 0.0210 | -0.0546 | 0.0210 |
|  | (0.0452) | (0.0830) | (0.0452) | (0.0830) | (0.0452) | (0.0830) | (0.0452) | (0.0830) |
| alt_a | -0.0361 | -0.0196 | -0.0361 | -0.0196 | -0.0361 | -0.0196 | -0.0361 | -0.0196 |
|  | (0.0417) | (0.102) | (0.0417) | (0.102) | (0.0417) | (0.102) | (0.0417) | (0.102) |
|  |  |  |  |  |  |  |  |  |
| Observations | 7,912 | 7,912 | 7,912 | 7,912 | 7,912 | 7,912 | 7,912 | 7,912 |

DCE results – conditional logit (split by experience)

| **Experience** | **10 yrs or less** | | **Greater than 10yrs** | |
| --- | --- | --- | --- | --- |
| **Attribute level** | **Co-eff (SE)** | **p-value** | **Co-eff (SE)** | **p-value** |
| **Risk level – base = all parents regardless of drinking levels** | | | | |
| Any risky drinking parent | 0.400 (0.14) | 0.005 | 0.459 (0.14) | 0.001 |
| Parents whose drinking impacts upon child/family | 0.709 (0.13) | 0.000 | 0.715 (0.13) | 0.000 |
| Parents whose drinking is the main safeguarding concern | 0.379 (0.08) | 0.000 | 0.264 (0.07) | 0.000 |
| **Who – base = parent only** | | | | |
| Parent and child | 0.097 (0.05) | 0.067 | 0.213 (0.05) | 0.000 |
| **When – base = during assessment phase** | | | | |
| During ongoing casework | 0.128 (0.05) | 0.015 | 0.136 (0.05) | 0.011 |
| **Length – base = 10 mins.** | | | | |
| 20 mins. | 0.310 (0.12) | 0.009 | 0.266 (0.12) | 0.026 |
| 40 mins. | 0.390 (0.11) | 0.000 | 0.228 (0.11) | 0.031 |
| 60 mins. | 0.349 (0.08) | 0.000 | 0.224 (0.08) | 0.003 |
| **Frequency – base = 1 session** | | | | |
| 2 sessions | 0.007 (0.11) | 0.949 | 0.162 (0.11) | 0.147 |
| 3 sessions | 0.076 (0.12) | 0.507 | 0.199 (0.11) | 0.075 |
| 6 sessions | 0.285 (0.08) | 0.000 | 0.116 (0.08) | 0.133 |
| **Content – base = leaflet** | | | | |
| Structured advice | 0.213 (0.14) | 0.120 | 0.329 (0.13) | 0.014 |
| Semi-structured discussion | 0.268 (0.13) | 0.035 | 0.323 (0.13) | 0.011 |
| Counselling | 0.217 (0.07) | 0.003 | 0.247 (0.07) | 0.001 |
| **Organisational support – base = discussed in supervision** | | | | |
| Supervision & organisational monitoring | -0.002 (0.05) | 0.968 | -0.010 (0.05) | 0.844 |
| **Training – base = half-day** | | | | |
| Full-day | -0.009 (0.05) | 0.855 | -0.091 (0.05) | 0.065 |
| Alternative A | -0.047 (0.05) | 0.318 | -0.048 (0.05) | 0.296 |
